# Decoding hand kinematics from population responses in sensorimotor cortex during grasping

**DOI:** 10.1088/1741-2552/ab95ea

**Published:** 2020-08-11

**Authors:** Elizaveta V Okorokova, James M Goodman, Nicholas G Hatsopoulos, Sliman J Bensmaia

**Affiliations:** 1 Committee on Computational Neuroscience, University of Chicago, Chicago, IL, United States of America; 2 Center for Bioelectric Interfaces, National Research University Higher School of Economics, Moscow, Russia; 3 Department of Organismal Biology and Anatomy, University of Chicago, Chicago, IL, United States of America; 4 Grossman Institute for Neuroscience, University of Chicago, Chicago, IL, United States of America

**Keywords:** brain machine interface, neuroprosthetics, bionic hand, dexterity, primary motor cortex, somatosensory cortex

## Abstract

*Objective*. The hand—a complex effector comprising dozens of degrees of freedom of movement—endows us with the ability to flexibly, precisely, and effortlessly interact with objects. The neural signals associated with dexterous hand movements in primary motor cortex (M1) and somatosensory cortex (SC) have received comparatively less attention than have those associated with proximal upper limb control. *Approach*. To fill this gap, we trained two monkeys to grasp objects varying in size and shape while tracking their hand postures and recording single-unit activity from M1 and SC. We then decoded their hand kinematics across tens of joints from population activity in these areas. *Main results*. We found that we could accurately decode kinematics with a small number of neural signals and that different cortical fields carry different amounts of information about hand kinematics. In particular, neural signals in rostral M1 led to better performance than did signals in caudal M1, whereas Brodmann’s area 3a outperformed areas 1 and 2 in SC. Moreover, decoding performance was higher for joint angles than joint angular velocities, in contrast to what has been found with proximal limb decoders. *Significance*. We conclude that cortical signals can be used for dexterous hand control in brain machine interface applications and that postural representations in SC may be exploited via intracortical stimulation to close the sensorimotor loop.

## 1. Introduction

The hand, a complex effector comprising dozens of degrees of freedom [1], allows us to flexibly, precisely, and effortlessly manipulate objects. The loss of hand function—as a consequence of spinal cord injury, for example—can have devastating consequences on quality of life [2]. In patients whose sensorimotor cortex is intact, some measure of independence can be restored with brain-machine interfaces (BMIs) that tap into the central neural pathways mediating manual dexterity [3]. Translating patterns of neural population activity into control signals for external devices is critical to advance such interfaces.

Reach-to-grasp movements have been traditionally decoded from cortical areas associated with the planning and execution of movement. Primary motor, premotor, and posterior parietal cortices have been the main targets of previous BMIs, yielding remarkable control of a robotic limb in both non-human primates [4–7] and human tetraplegic patients [8–11]. However, the focus of most decoding studies has been on reaching movements performed by the proximal limb (elbow and shoulder) [12, 13]. The distal limb (wrist and finger joints), critical to object interactions, has received comparatively little attention (but see [14]) primarily due to the greater complexity of manual behavior [15] and the difficulty of simultaneously tracking tens of hand joints [16].

A critical complement to neural signals involved in controlling movements are signals responsible for conveying sensory feedback about the consequences of those movements [17]. Indeed, the motor apparatus receives continuous proprioceptive feedback from joints and muscles that signal the position of body in space, the forces it exerts, and mediates error-corrective motor adjustments [18]. Proprioceptive impairments lead to major deficits in motor behavior, leading to slow, effortful, and imprecise movements [19–21]. Two cortical fields in somatosensory cortex (SC)—Brodmann’s areas 3a and 2 [22, 23]—contain neurons that respond to active and passive manipulation of joints and muscles [24–26], to forces applied by the muscles, and to torques applied to the joints [27]. However, the vast majority of previous studies of proprioceptive representations in SC have focused on the proximal limb, particularly during reaching movements.

Hand-related responses of *individual* SC neurons have been characterized during passive deflections of the hand joints [26, 28] and during active hand movements [29]. However, the degree to which hand movements and postures are encoded across *populations* of SC neurons has not been investigated. One way to address this question is by assessing our ability to decode hand kinematics from the responses of populations of SC neurons. Earlier decoding studies focused on the transport component (i.e. reaching), which primarily involves the proximal limb [30–32]. Those that incorporated distal-limb movements either attempted to classify a small set of discrete hand postures [33] or to decode (1-dimensional) continuous grip aperture or grip force [34]. To the extent that somatosensory neurons carry detailed information about hand movements, these neural representations might be exploited to convey artificial proprioceptive feedback through intracortical microstimulation (ICMS) [35–37], paralleling efforts to convey artificial tactile feedback through ICMS [38, 39].

The goal of the present study is to assess the degree to which hand kinematics can be decoded from the responses of populations of sensorimotor neurons, the main novelty being the attempt to decode hand state from SC. To this end, we trained two monkeys to grasp 35 objects of varying sizes, shapes and orientations, while tracking their time-varying hand kinematics using a camera-based motion tracking system. We simultaneously recorded the responses in the hand representations of M1 and SC using chronically implanted electrode arrays. We then inferred object identity (referred henceforth as ‘classification’) as well as continuous joint kinematics (referred henceforth as ‘decoding’) from neural signals while the hand preshaped to grasp an object (before contact). First, we show that neural signals in both M1 and SC carry faithful representations of the hand by using these signals to classify objects before they are grasped or to reconstruct time-varying joint trajectories. Second, we show that different cortical fields in M1 and SC carry different amounts of information about hand state. Finally, we find that we can better decode posture than movement, in contrast to what has been shown for motor representations of the proximal limb, suggesting possible differences in cortical encoding for proximal and distal limb. Our results underscore the promise of using M1 signals to achieve control of the hand during grasping in a BMI setting and demonstrate that SC populations carry a faithful representation of time-varying hand configuration that could in principle be exploited to restore proprioception through ICMS.

## 2. Methods

### 2.1. Animals and surgery

We took recordings from two male Rhesus macaques ranging in age between 6 and 15 years and weighing between 8 and 11 kg. Monkey 2 was implanted twice (monkey 2a and 2b) with an interval of 2 years between recording periods and using different recording techniques. All animal procedures were performed in accordance with the rules and regulations of the University of Chicago Animal Care and Use Committee. Monkeys received care from a full-time husbandry staff, and a full-time veterinary staff monitored animals’ health.

Surgical procedures consisted of implantation of a head-fixing post onto the skull, craniotomy, implantation of a sealed recording chamber (monkey 2a), and implantation of Utah electrode arrays (UEAs, Blackrock Microsystems, Inc. Salt Lake City, UT, monkeys 1 and 2b) and floating microelectrode arrays (FMAs, Microprobes for life science, Gaithersburg, MD; monkey 1) or of semi-chronic Microdrive electrode arrays (Gray Matter Research, Bozeman, MT [40, 41]; monkey 2a). All procedures were performed under aseptic conditions and anesthesia induced with ketamine HCl (20 mg kg^–1^, IM) and maintained with isoflurane (10–25 mg kg^–1^ per hour, inhaled).

### 2.2. Behavioral task

**Figure 1.**
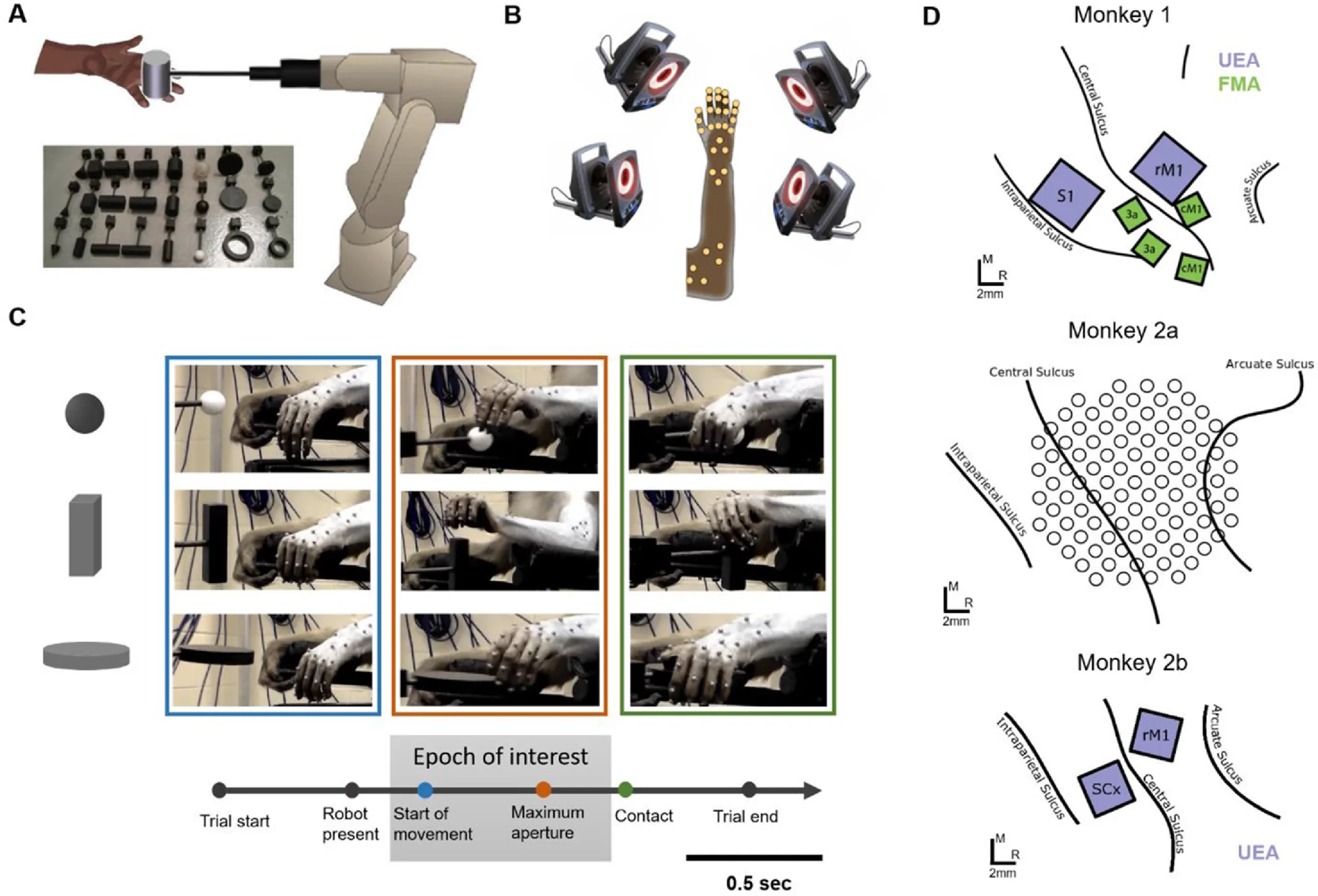
Behavioral task and experimental set-up. (A) Experimental apparatus. On each trial, a robotic arm presented the animal one of 35 objects (inset, supplementary figure 1 and supplementary table 1). (B) Motion tracking. We placed 30 reflective markers on the animal’s joints and tracked their 3D position with a 14-camera Vicon motion tracking system. For joint names and locations, see supplementary figure 2. (C) Trial structure. We focused our analysis on the interval from the start of monkey’s hand movement until just before contact with the object. Images show hand postures at the start of movement (blue box), at maximum aperture (orange box), and during grasp (green box) for three example objects. (D) Array placement: Utah electrode arrays (UEA, purple, monkey 1 and 2b), floating microelectrode arrays (FMA, green, monkey 1), and Gray Matter array (monkey 2a). For sample sizes, see supplementary table 2. R: rostral, M: medial.

We trained monkeys to grasp 35 objects varying in shape, size, and orientation to induce a wide range of hand postures (figure 1(A), inset and supplementary figure 1) (available online at stacks.iop.org/JNE/17/046035/mmedia). Throughout the session, the head-fixed monkey sat in a chair facing a three DoF robotic arm (figure 1(A), top). The arm of the monkey rested on the cushioned armrest and remained largely immobile during the grasp. At the beginning of each trial, an object was attached to the robotic arm using a weak magnet and presented to the animal. The monkey’s task was to grasp the object and exert enough grip force so that, when the robot retracted, the object would be disengaged from its magnetic coupling with the robot and remain in the monkey’s hand (figure 1(C)). Animals were trained to keep their elbow on the armrest to minimize movements of the proximal limb. Motionlessness of the proximal limb was enforced by a photosensor embedded into the armrest that triggered an early, unrewarded end to a trial if the arm was lifted to expose it to light.

### 2.3. Kinematics

We recorded hand and elbow kinematics using a camera-based motion tracking system (Vantage, VICON, Los Angeles, CA). To this end, we placed 30 reflective markers on the joints of the hand, wrist, and proximal limb (figure 1(B), supplementary figure 2). Ten cameras were used to capture the kinematics of the first monkey at a rate of 250 Hz, and fourteen cameras were used to capture the kinematics of the second monkey at a rate of 100 Hz. We then labeled each marker using Nexus software (VICON, Los Angeles, CA) and performed inverse kinematics (OpenSim [42]) from the resulting time-varying marker positions to infer time-varying joint angles of the limb (22 DoFs for monkey 1 and 30 DoFs for monkey 2). Joint angles were smoothed with a 50-ms moving average and the angular velocities were computed from these. For each trial, we identified the start of movement (finger and wrist), maximum aperture of fingers, and contact with an object (finger and palm). For monkey 1, we labeled these events manually for all trials. For monkey 2, we only labeled a subset of trials and then used joint angular kinematic trajectories spanning 200 ms before and after each frame as features to train a multi-class linear discriminant classifier to discriminate among 6 classes: all events of interest and ‘no event’. The log likelihood ratio was used to determine which of the events was more probable relative to ‘no event’. Additional constraints were imposed to ensure that start of movement preceded maximum aperture and maximum aperture preceded grasp. Among all sessions of monkey 2, the mean and the standard error of the model deviation from hand-labeled events were 16 ± 9.5 ms for start of finger movement, 21 ± 8.8 ms for start of wrist movement, 11 ± 8.8 ms for maximum aperture, –25 ± 9.7 ms for palm contact with an object, and –48 ± 12.1 ms for finger contact with an object.

Across trials and objects, the mean interval ± standard deviation between the start of wrist movement and maximum aperture was 524 ± 164 ms, 466 m ± 70 ms and 426 ± 110 ms and that between maximum aperture and first contact with fingers was 273 ± 115 ms, 447 ± 24 ms, 114 ± 43 ms, for monkeys 1, 2a and 2b, respectively.

### 2.4. Electrophysiology

We recorded neural signals from monkeys 1 and 2b using UEAs placed in the pre- and post-central gyri and, for monkey 1, from FMAs placed in the posterior and anterior banks of the central sulcus (figure 1(D)). For monkey 2a, we recorded neural signals using arrays of depth-adjustable electrodes (SC96) positioned over the central sulcus (figure 1(D)). We used offline spike sorting (Offline Sorter, Plexon, Dallas, TX) to remove non-spike threshold crossings and isolate individual units in the high-pass filtered signal.

Motor units recorded from the crest of the precentral gyrus were classified as rostral M1 (rM1), whereas those recorded from the depth of the anterior bank of the central sulcus were classified as caudal M1 (cM1) [43]. The provenance of somatosensory units was defined by anatomical location and manual functional mapping prior to recordings (as previously described in [29]).

### 2.5. Neural data preprocessing

Time-varying firing rates of all recorded neurons were computed by summing all events in 10-ms bins. We then soft-normalized the firing rates (divided by their range plus a small increment) and convolved the resulting rates with a Gaussian kernel with a standard deviation of 15 ms.

### 2.6. Session stitching

To achieve a sufficient sample size from each cortical field, we pooled data from all sessions for each monkey (supplementary table 2). To this end, we first aligned the kinematics from each condition (object) to maximum hand aperture. Then, we eliminated trials on which the animal adopted a grasping strategy for a given object that was different from that used for that same object on other trials to ensure that the pooled neuronal responses were associated with similar kinematics. Specifically, we discarded trials on which any one time-varying joint angle trajectory exhibited a correlation with its time-varying trial-averaged angle that was lower than 0.7. The remaining kinematics were consistent enough from trial to trial to warrant averaging across trials. We then used the average kinematic traces from 600 ms before maximum aperture to just before finger contact with an object, estimated as an average time to finger contact within each monkey (273, 447 and 114 ms for monkeys 1, 2a, 2b, respectively).

### 2.7. Classification

To assess the degree to which grasping different objects involved different patterns of kinematics and neuronal activity, we attempted to classify the 35 objects using either kinematics or neural data. Specifically, we used multiclass linear discriminant analysis, where the independent variables were the mean joint position or neuronal activity over an interval spanning 150 ms before finger contact with the object, when the hand had nearly reached its final posture before grasp. We trained the classifier using all but one randomly selected trial from each class (object) and tested it on the left-out trial. We performed the random trial selection 10 times and reported mean performance across folds.

### 2.8. Decoding

For continuous decoding, we fit the standard Kalman filter to all recorded joints. The Kalman filter comprises two estimates of some variable, ${x_t}$, which in our case was a joint angle or joint angular velocity at some time t. These estimates are:

\begin{equation*}\hat x\,_t^k = A{x_{t - 1}} + {q_t}\end{equation*}

\begin{equation*}\hat x\,_t^n = B{z_t} + {w_t}\end{equation*}

where $\hat x\,_t^k$ is the estimate of ${x_t}$ based on past kinematics; A is a state transition matrix; ${q_t}$ is a Gaussian process with zero mean and covariance matrix Q; $\hat x\,_t^n$ is an estimate of ${x_t}$ based on current neural activity; ${z_t}$ is observed spiking activity with accumulated lags/leads over interval $\tau \in \left[ { - 150,\,150} \right]\,{\text{ ms}}$ for acausal filters and $\tau \in \left[ { - 150,\,0} \right]\,{\mathrm{ms}}$ for causal ones, in steps of 30 ms; B is an observation model matrix; and ${w_t}$ is a Gaussian process with zero mean and covariance matrix W. We used a fusion algorithm to obtain a single best estimate of ${x_t}$ for each time t (see details in [44, 45]).

To obtain filter parameters (A,B,Q,W), we fit the model using 80% of randomly selected trials, validated the hyperparameters on half of the remaining trials (10%), and gauged model performance on the other half (10%). We used L2-norm regularization, which penalizes the L2-norm of matrix B by an amount determined by a hyperparameter, λ. Performance was assessed using the coefficient of determination (${R^2}$). To compare our results to previous ones, we also computed Pearson’s correlation and the normalized Root Mean Squared Error (rRMSE), prediction error expressed as a proportion of the joint’s range of motion (see [14] for details and supplementary figure 3).

### 2.9. Optimal single-lag latency

To find the optimal asynchrony between kinematics and neural responses, we tested the model with neural data shifted relative to kinematics to different degrees (at +-250 ms, +-200 ms, +-150 ms, +-90 ms, +-50 ms, +-3 ms, +-2 ms, +-1 ms, 0 ms) using a single-lag model and a causal filter (half Gaussian kernel). We then found the lag or lead at which cross-validated performance was highest for each joint. We used this lag/lead across all joints for each single-lag model. Asynchronies were optimized separately for posture and movement.

### 2.10. Controls

In a Kalman filter, the autocorrelation in the observed process is captured by matrix A in equation (1). To control for the possibility that kinematics were predictable from their past state alone, we randomly shifted spikes within each trial (preserving the spike count) and recomputed decoding performance. In addition, we compared the standard Kalman filter decoder to other types of linear and non-linear decoders, including Wiener Filter, Wiener Cascade Filter, Extreme Gradient Boosting, Dense Feedforward Neural Network, Recursive Neural Network, Gated Recurrent Unit, and Long Short Term Memory Network described in detail in [32] (supplementary figure 4).

## 3. Results

### 3.1. Complexity of the task and associated neuronal responses

**Figure 2.**
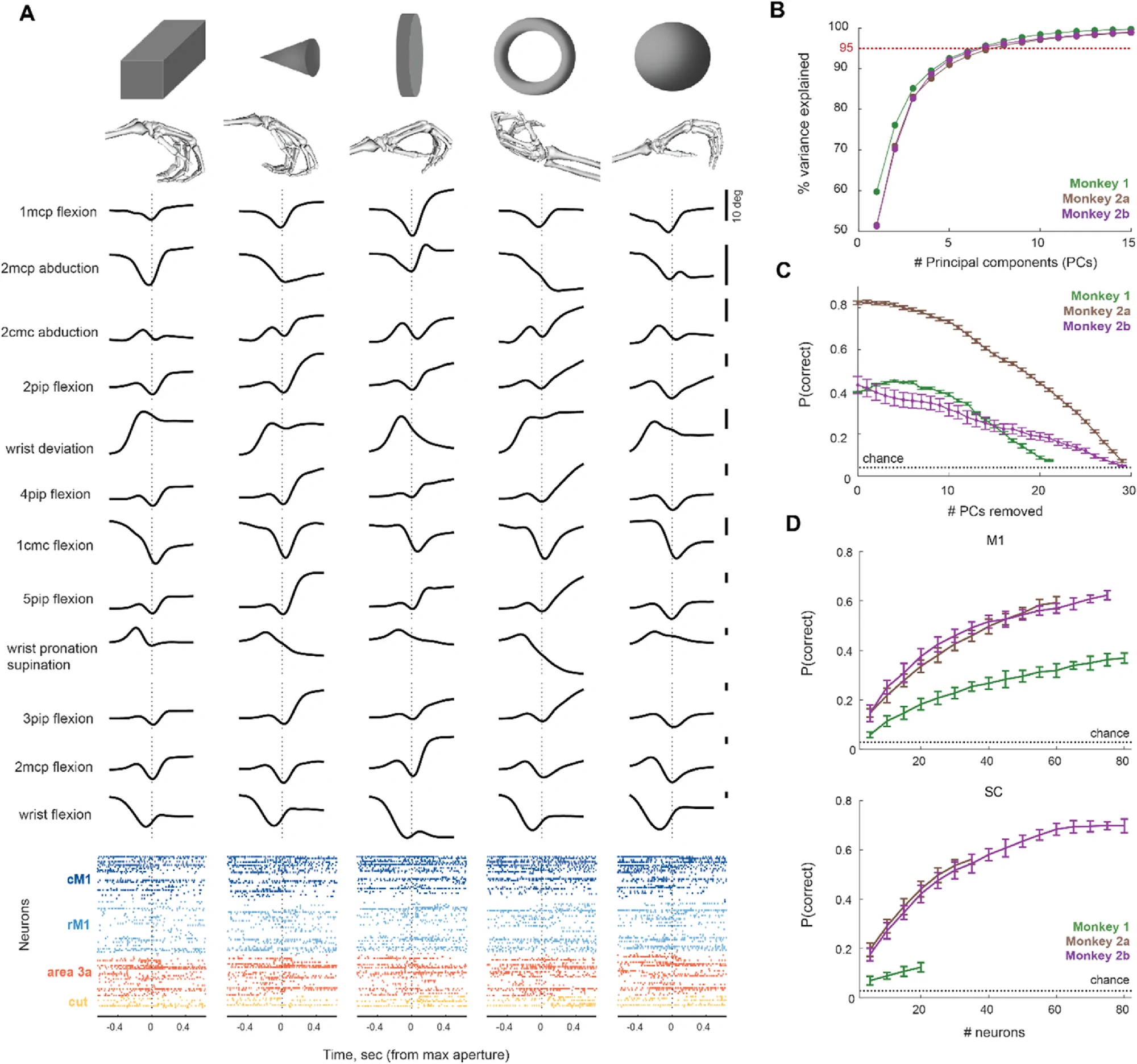
*Grasping objects of different shapes elicits varying kinematics and neural responses*. (A) Example hand postures just before contact with corresponding mean kinematic trajectories of the 12 joints that move the most along with the evoked spiking activity of populations of neurons pooled from all sessions from caudal M1 (dark blue, N = 24), rostral M1 (light blue, N = 32), area 3a (orange, N = 25), and cutaneous neurons from areas 1 and 2 (yellow, N = 6) for 5 grasped objects (medium horizontal block, point, large outward disk, vertical ring and large sphere, see supplementary figure 1 for details). Trials were aligned to maximum aperture of the hand (t = 0). Hand nomenclature is described in supplementary figure 2. (B) Cumulative percentage of variance vs. number of principal components of kinematics for the two monkeys. (C) Classification of objects based on kinematics projected on a decreasing number of principal components (the abscissa shows the number of removed components, ranked by variance) for all monkeys. Dotted horizontal line indicates probability of randomly selecting an object (1/35). Error bars denote the standard error of the mean classification performance across sessions. (D) Classification of objects based on neural signals (top—M1, bottom—SC) as a function of neuronal sample size. Error bars denote the standard error of the mean.

First, we wished to characterize the complexity of the grasping behavior—to what extent did the animal produce different hand movements when grasping different objects?—and of the associated neuronal responses in sensorimotor cortex. The large and varied set of objects elicited a variety of hand conformations and neural responses in M1 and SC (figure 2(A)). To characterize the complexity of the task, we first performed principal component analysis (PCA) on the hand kinematics and found that only 7–8 principal components explain most (95%) of its variance (figure 2(B)). We then assessed whether the remaining components carry any information about the task. To that end, we projected kinematics on a subset of PCs, removing PCs in decreasing order, and inferred the object identity with projected kinematics. We found that even after subtracting the first eight PCs, we were still able to classify objects well above chance (figure 2(C))(cf [46]). Classifier performance was tightly linked to how different the kinematics were for different objects—more stereotyped grasps led to poorer classification performance as expected (supplementary figure 5). These results indicate that the monkeys produced a variety of hand configurations in an object-dependent way.

We then asked whether these object-dependent differences in kinematics were reflected in the neuronal responses. We found that in all monkeys as few as 5 neurons in a population were enough to achieve above chance classification performance. Performance increased steadily with population size in both M1 and SC (figure 2(D)).

### 3.2. Decoding single-trial kinematics from M1 and SC signals

**Figure 3.**
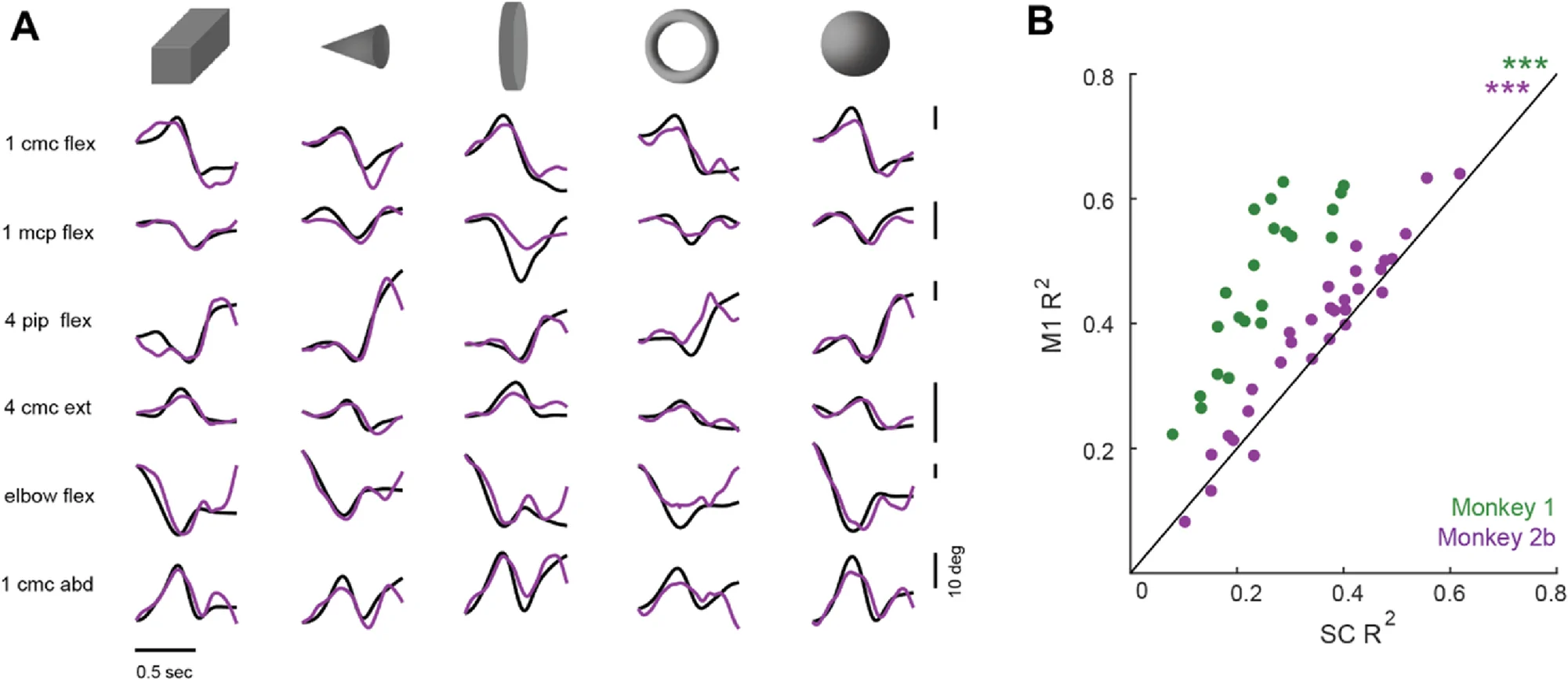
*Decoding single-trial kinematics from M1 and SC signals.* (A) Six joint angle trajectories produced during one grasp of each of five objects (black) along with the same trajectories decoded from the responses of 36 M1 neurons in monkey 2b (purple). Hand nomenclature is described in supplementary figure 2. (B) Performance of single-trial kinematics decoder for one session each from two monkeys with the same number of neurons in M1 and SC (N = 15 and 36 for monkeys 1 and 2b, respectively). Each dot corresponds to the mean decoding performance for one joint averaged over 5 cross-validation folds with randomly selected neurons from the recorded population. Stars indicate significance of Wilcoxon Signed Rank Test for matched samples: ***—alpha level of 0.001. Only sessions with a sufficient number of simultaneously recorded units were used in this analysis (monkeys 1 and 2b).

Next, we assessed the degree to which the responses of neuronal populations in M1 and SC convey information about time-varying hand kinematics. We found that we could accurately decode single-trial kinematics of most joints in sessions for which we had enough simultaneously recorded neurons (average performance across joints: *R^2^*= 0.52 for 44 M1 neurons in monkey 1, *R^2^* = 0.43 and 0.39 for 36 M1 and 37 SC neurons, respectively, in monkey 2b; figure 3(A), supplementary figure 3). Furthermore, M1 decoders significantly outperformed SC ones (figure 3(B)).

To assess the degree to which performance was decoder dependent, we implemented 8 other linear and non-linear decoders (supplementary figure 4). We found that performance was very similar across decoders and thus only report performance of the Kalman Filter in the main text given its simplicity and prevalence.

As expected, performance was slightly poorer if we constrained the decoder to be causal, as is required in online applications (supplementary figure 6(A)).

### 3.3. Decoding kinematics averaged across trials from M1 and SC signals

We recorded data over many sessions that individually did not yield enough neural signals to decode single-trial kinematics. To expand our data set, we pooled neural responses across all sessions from each monkey and used these pooled responses to decode the kinematics trajectory for each object, averaged across trials and sessions (see Methods). Importantly, we verified that, for sessions with sufficient sample size, decoding performance for single-trial and mean kinematics was comparable and yielded similar conclusions about the kinematics representations in M1 and SC (supplementary figure 6(B)). We then compared decoding performance of trial-averaged kinematics from M1 and SC using these neuronal responses pooled across sessions. For monkeys 1 and 2b, like their single-trial counterparts, trial-averaged decoders based on M1 responses tended to outperform those based on SC responses (supplementary figure 6(C), Wilcoxon Signed Rank Test for matched samples: Z = 4.0 and 4.5 for monkeys 1 and 2b, respectively, *p* < 0.0001. However, for monkey 2a, the reverse was true (Z = –4.4, p < 0.0001). The difference between monkeys is likely due to the respective locations of SC recordings (area 3a vs. area 2; see supplementary table 2 for sampling of cortical fields).

### 3.4. Comparing cortical areas

**Figure 4.**
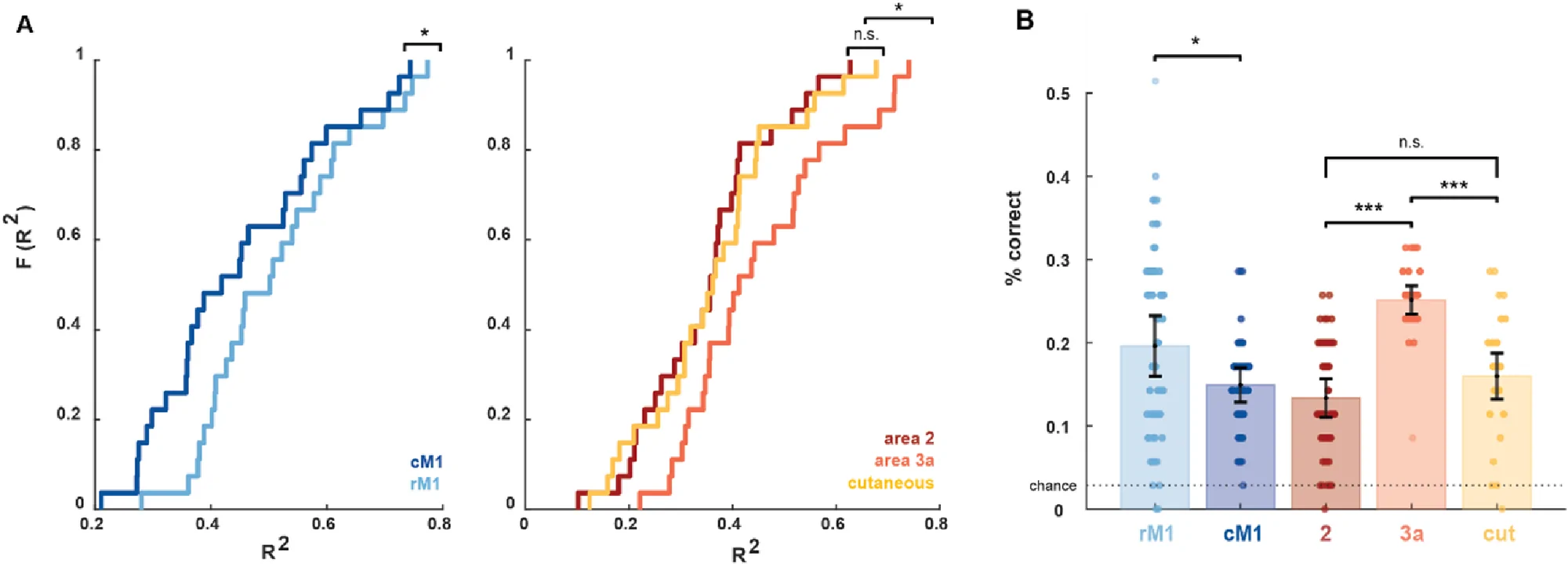
Comparison of cortical fields. (A) Left: cumulative distribution of the accuracy (R^2^) with which trial-averaged kinematics for all joint angles were decoded based on rM1 and cM1 responses. Right: Cumulative distributions of trial-averaged decoding accuracy for individual joint angles based on different cortical fields of SC. (B) Performance of an object classifier based on neuronal responses for monkey 2 (including both 2a and 2b) with each cortical area assessed separately. Each point indicates classification performance for one cross-validation fold. Stars in all plots indicate significance of a one-sided Mann-Whitney U test: n.s.—not significant, *—alpha level of 0.05 ***—alpha level of 0.001. Sessions from monkey 2 (a and b) were pooled together for this analysis. We discarded monkey 1 since we did not have sufficient number of units from all subfields shown here.

Next, we performed a more detailed analysis of how information about hand posture is distributed within M1 and SC (figure 4). Given the small samples collected from each cortical field in a given session, we pooled data from each monkey across sessions and decoded trial-averaged kinematics, having verified that this approach does not introduce systematic biases (supplementary figure 6(B)).

The caudal region of M1 contains more corticomotoneuronal (CM) cells—which make monosynaptic connections with motoneurons—than does its rostral counterpart, and these CM neurons are thought to be critical for highly skilled movements, particularly of the hand [43]. When comparing performance between areas (keeping the sample size equal), however, we found that decoding based on signals from rostral M1 systematically outperformed that from caudal M1 signals (one-sided Mann-Whitney U test: Z = 1.83, p = 0.033) (figure 4(A), left), thereby violating our expectations.

While neurons in Brodmann’s area 3a exhibit almost exclusively proprioceptive responses, those in area 2 exhibit mixed proprioceptive and cutaneous responses [47]. Cutaneous responses to skin stretch caused by joint movement may interfere with proprioceptive representations of the hand or contribute to them. We found that populations of neurons in area 3a yielded significantly better performance than did proprioceptive neurons in area 2 and neurons in areas 1 and 2 that are classified as cutaneous during hand mapping (one-sided Mann-Whitney U test: area 3a vs area 2, Z = 2.3, p = 0.0107; area 3a vs cutaneous, Z = 1.95 p = 0.0253; area 2 vs cutaneous, Z = 0.329, p = 0.37) (figure 4(A), right).

We also assessed our ability to classify objects based on the responses evoked in different cortical populations and found results to be consistent with those of our kinematics decoding analysis: rM1 was more informative about object identity than was cM1 and area 3a was more informative about object identity than were areas 1 and 2 (figure 4(B)).

### 3.5. Decoding joint groups

**Figure 5.**
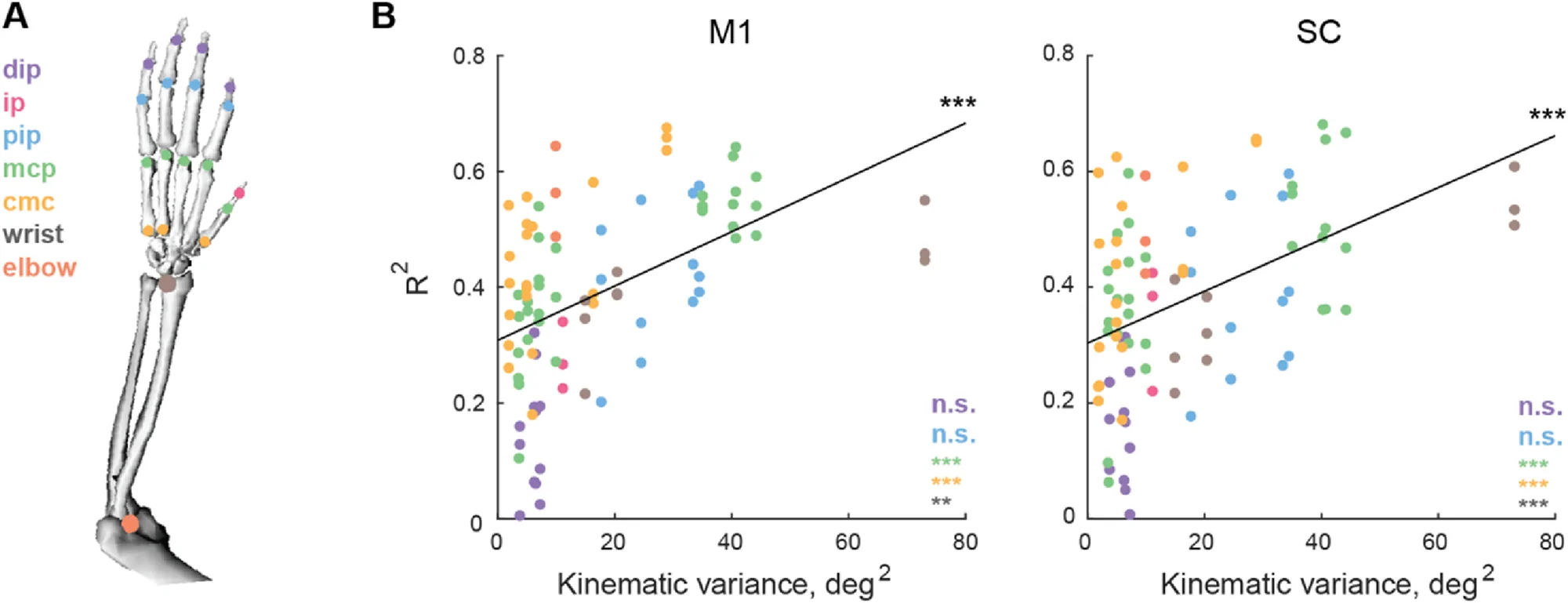
Decoding performance broken down by joint groups. (A) Location of joints color-coded by group: distal (dip, purple), proximal (pip, blue), interphalangeal (ip, pink), metacarpophalangeal (mcp, green), carpometacarpal (cmc, yellow), wrist (brown), elbow (orange). (B) Single joint decoding performance color-coded according to the scheme in A as a function of kinematic variance for all sessions of monkey 2b using M1 (N = 16 in each session) and SC (N = 19) populations. Each point denotes the mean over 5-fold cross-validation of each DoF. Black lines show best linear fit between kinematic variance and decoder performance of all DoFs. Stars indicate significance of correlation coefficient color-coded by joint group: ***—alpha level of 0.001, **—alpha level of 0.01, n.s.—not significant. Only sessions with a sufficient number of simultaneously recorded units in both areas were used in this analysis (monkey 2b). Monkey 1 was discarded due to incomplete set of recorded joints.

Next, we investigated whether the sampled subpopulations of M1 and SC preferentially encoded movements of different portions of the hand. Indeed, both M1 and SC are somatotopically organized [48–52], so incidental electrode placement might have led to better decoding for some hand regions than others. With this in mind, we divided the joints into 7 groups—distal, proximal, interphalangeal, metacarpophalangeal, carpometacarpal hand joints, wrist joints (pronation-supination, flexion, extension), and proximal arm joints (elbow)—and assessed the average decoding performance for each group (figure 5(A)). With the exception of a few distal hand joints, all joint groups were decoded well, and decoding accuracy improved as the joint’s range of motion increased (*r* = 0.5, *p* = 5.3 × 10^-7^ for M1 and *r* = 0.46, *p* = 5.1 × 10^-6^ for SC; figure 5(B)). Significant positive correlations with motion variance were observed within metacarpophalangeal (*r* = 0.79, *p* = 2.4 × 10^-7^ for M1 and *r* = 0.53, *p* = 0.0025 for SC), carpometacarpal (*r* = 0.63, *p* = 0.002 for M1 and *r* = 0.64, *p* = 0.002 for SC) and wrist (*r* = 0.74, *p* = 0.024 for M1 and *r* = 0.88, *p* = 0.0016 for SC) joint groups, but not within distal (*r* = 0.16, *p* = 0.62 for M1 and *r* = –0.07, *p* = 0.83 for SC) or proximal (*r* = 0.36, *p* = 0.25 for M1 and *r* = 0.15, *p* = 0.63 for SC) joints. These observations suggest that the arrays impinged upon neural populations that spanned the representation of the entire hand in both M1 and SC and that decoding performance was partially dependent on the range of joint motion.

### 3.6. Decoding kinematic synergies

**Figure 6.**
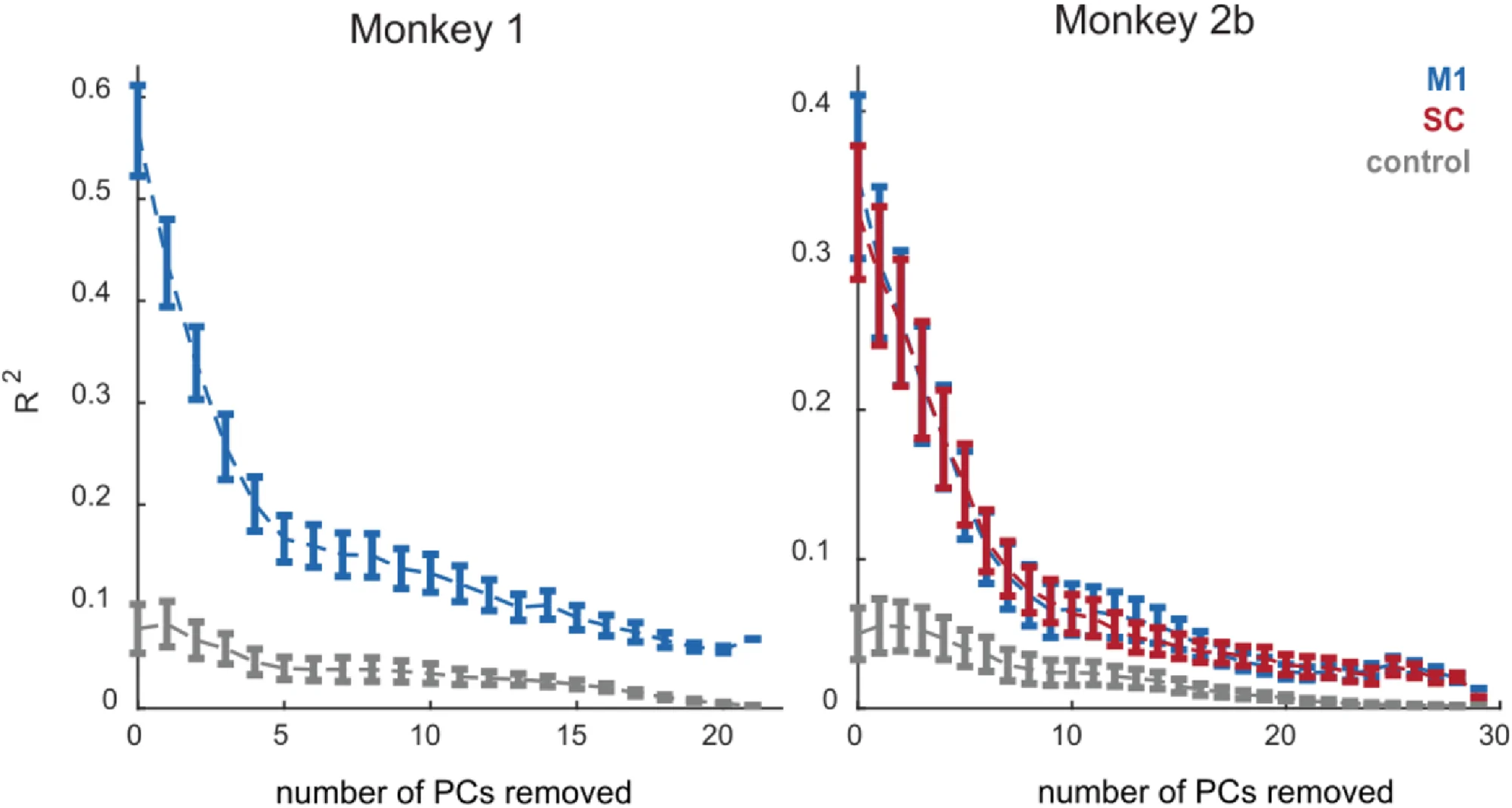
Decoding synergistic movements. Average performance of decoding kinematics projected on decreasing number of principal components (x axis represents the number of removed components, ranked by variance) using populations of M1 (blue) and SC (red) neurons. Grey bars show decoding performance with randomly shifted spikes. Error bars denote the standard error of the mean. Only sessions with a sufficient number of simultaneously recorded units were used in this analysis (M1 and SC in monkey 1 and M1 only in monkey 2b).

Joint kinematics of the hand have been shown to exhibit systematic correlational structure, with some joints tending to move together. One possibility is that only patterns of highly correlated joint movements can be decoded from the neuronal activity in M1 and SC and that more subtle aspects of hand movements cannot. To this hypothesis, we performed a principal component analysis (PCA), projected kinematics onto a sequentially reduced sets of principal components (PCs), removing PCs in decreasing order of eigenvalue (i.e. higher-variance components first). We then decoded kinematics in this reduced space from neuronal signals in M1 and SC. Chance performance was computed by randomly permuting the spike times within each trial to distort the temporal structure of the neuronal response, predictably yielding poor performance. We found that higher order (low-variance) components could still be decoded above chance (figure 6), despite the fact that 95% of the variance in the kinematics can be explained with only 7–8 principal components (figure 2(B)). Note that the trajectories projected onto higher order components of kinematics are still object dependent and thus under volitional control (figure 2(C))[46].

### 3.7. Decoding postures vs. movements

**Figure 7.**
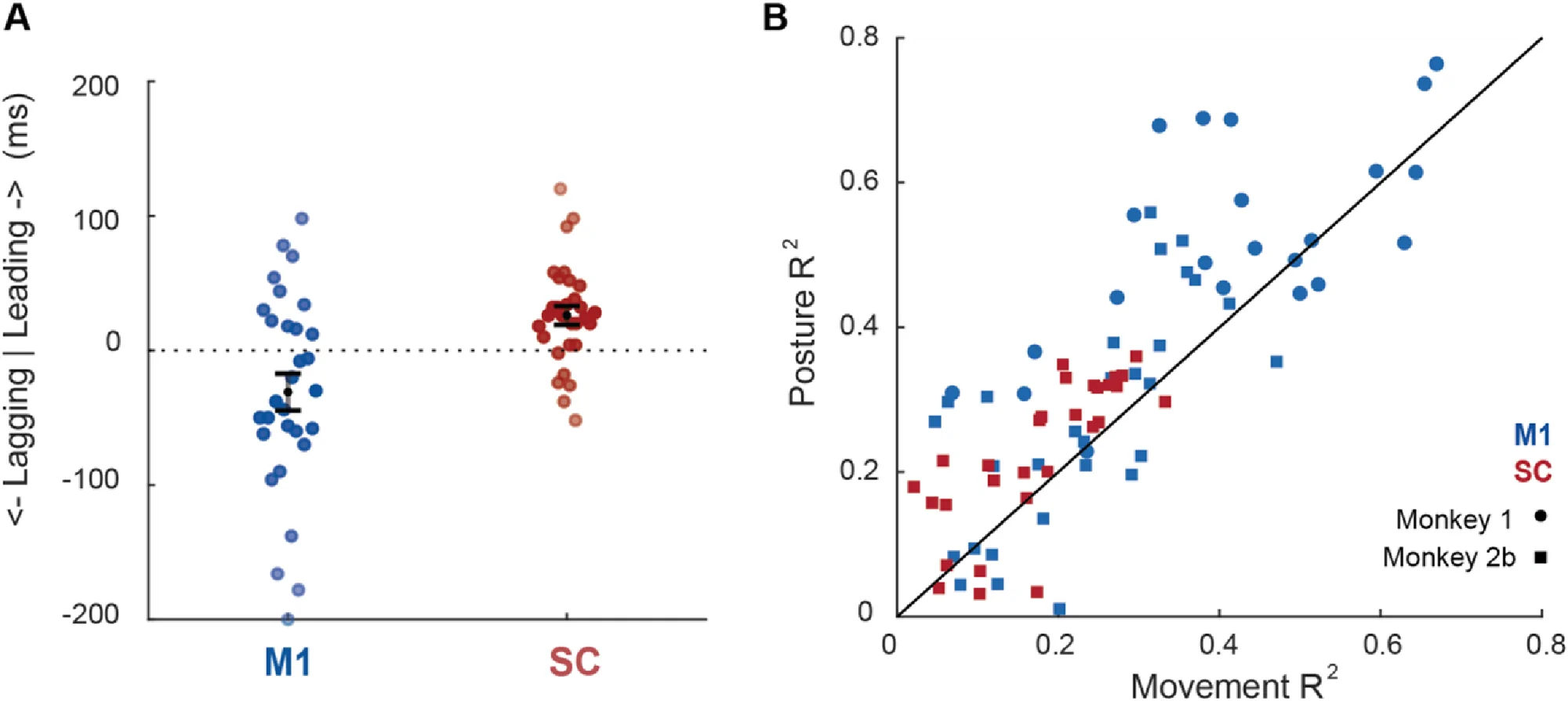
*Posture vs. movement decoding.* (A) Optimal neural latencies for each joint of monkey 2b for M1 (blue) and SC (red). Error bars indicate mean and standard errors of the mean of latencies for each area. ‘Leading’ denotes more sensory-like neurons, where the kinematics lead the neuronal response. (B) Decoding performance of posture (*y*-axis) and movement (*x*-axis) decoding for a randomly selected population of 36 M1 (blue) or SC (red) neurons. Each marker indicates performance for one joint averaged over 5 folds. Different marker symbols denote different monkeys. Only sessions with a sufficient number of simultaneously recorded units were used in this analysis (M1 and SC in monkey 2b and M1 only in monkey 1).

In the above analyses, we showed that time-varying postures could be directly decoded from neuronal activity. This approach stands in contrast to that adopted for proximal limb kinematics or the application of proximal limb-related M1 activity to cursor control, which typically involve decoding joint or endpoint velocities from neuronal responses and then integrating these to obtain postures [6, 11, 53–55]. Indeed, even SC decoders for reaching movements appear to perform better when decoding limb velocity than limb posture [30]. With this in mind, we assessed whether neuronal responses in M1 and SC preferentially encode postures or movements during grasp. To this end, we reconstructed joint angular velocities from sensorimotor responses and compared these to our reconstructions of angular positions. For this analysis, we used a single latency—determined to yield peak performance (for posture and movement separately within each area)(figure 7(A))—because multi-lag models allow for integration (from a velocity to a position signal) or differentiation (from a position to a velocity signal), thereby obscuring the distinction between postural and movement coding [29]. Using 20 units from each cortical subpopulation, we found that postures could be significantly better reconstructed than could movements (figure 7(B)) (Wilcoxon Signed Rank Test for matched samples: Z = 2.91 and 2.01, p = 0.004 and 0.044, for M1 in monkeys 1 and 2b, respectively; Z = 3.42, p = 0.0006 for SC in monkey 2b) in contrast to what has been observed for the proximal limb [11, 30, 56].

## 4. Discussion

### 4.1. High dimensional decoding

Most decoding studies to date have focused on continuous movement of a few degrees of freedom, usually including shoulder and elbow [4, 5, 7, 13, 57–59] and, less frequently, wrist [8, 9] and finger joints [14, 60]. Because of the complexity of the space of hand kinematics, most previous efforts to decode hand postures were either discrete, focusing on classification of a finite number of finger-wrist configurations [33, 61–63] or limited to a few common continuous finger movements, such as pinch, scoop, grip, and whole finger flexion/extension, and whole-hand aperture [8, 9, 64, 65]. As new hand tracking technologies are becoming available [16, 66, 67], decoding tens of joints simultaneously is becoming increasingly manageable [14]. Here, we show that up to 30 degrees of freedom can be decoded with a relatively small population of sensorimotor neurons even with a simple linear decoder.

### 4.2. Decoding from M1

Our approach is similar to that described in [14], in which 27 degrees of freedom of grasping kinematics were reconstructed from the responses of neurons in posterior parietal cortex and M1. Decoders built from the one area the two studies have in common, M1, yielded comparable performance (supplementary figure 3).

Anatomically, M1 can be divided into two regions: rostral and caudal M1. A large fraction of neurons in the caudal part of M1 make direct connections with motoneurons in the spinal cord and might be particularly relevant for dexterous hand control whereas neurons in the rostral region comprises a larger fraction of neurons that contact mainly spinal interneurons and only indirectly drive muscles [43]. Counter to predictions derived from the anatomy, we found that decoders built with signals from rostral M1 actually outperformed decoders built with signals from caudal M1. Because grasp is so habitual and involves highly correlated patterns of joint movements, it may not constitute an ‘expert’ behavior and thus may not require a direct line to muscles. Had the animals been performing non-prehensile dexterous hand movements, we might have found an advantage of caudal M1 [68].

### 4.3. Decoding from SC

Previous attempts to decode kinematics from SC activity focused on proximal limb movements. Decoding reaching movements from combined M1 and SC activity yielded better performance than that achieved using M1 signals alone [5, 34]. Decoding limb kinematics using electrocorticographic (ECoG) signals from SC achieved similar performance as with ECoG signals from M1 [31, 33].

In the present study, we decode, for the first time, hand kinematics from the spiking activity of neurons in Brodmann’s areas 3a and 2 and find that both areas yield performance well above chance for all degrees of freedom. Area 3a showed performance comparable to M1, consistent with earlier observations that single-unit responses in areas 3a and 4 are tightly linked to time-varying hand postures [29]. Area 2, which lies downstream of area 3a but also receives cutaneous input, yielded significantly worse performance than did M1, consistent with the hypothesis that the cutaneous input to this area obscures the proprioceptive representations, even before contact. While movement has been previously shown to activate cutaneous neurons in the absence of contact [47, 69], our results suggest that cutaneous signals obscure rather than complement muscle- and tendon-derived signals about hand posture.

### 4.4. Posture and movement decoding

Neurons in motor cortex and proprioceptive areas of somatosensory cortex have been shown to preferentially encode velocity of the proximal limb (movement), rather than its position (posture) [56, 70]. Consistent with this finding, decoders of velocities generally outperform those of joint positions both online [11] and offline [30, 56]. However, this preferential encoding and decoding of movement over posture has been tested exclusively for the proximal limb. When we directly compared posture and movement decoding of the hand, we found that the former can be more faithfully decoded than can the latter. Importantly, for this analysis, we restricted the decoder to a single lag. Indeed, multi-lag decoders allow for linear integration of movement signals which converts these into postural signals. As might be expected, then, the difference in performance between posture and movement decoders is less pronounced for multi-lag models compared to their single-lag counterparts (supplementary figure 7) and multi-lag decoders sometimes lead to an advantage for postural decoding even in the proximal limb [34]. Postural preference in our analysis implies a difference between proximal and distal limb representations, which may be inherited from the different inertial and biomechanical properties of the arm and the hand and is well suited to support stereognosis [29].

### 4.5. Decoding methods

The application of machine learning to kinematic/cursor decoding is becoming standard practice [32]. However, the extent to which recently developed decoding approaches robustly improve performance of high dimensional decoders (of hand kinematics, e.g.) has not been systematically investigated. Here, we applied a variety of linear and non-linear approaches to decoding hand movements (described in detail in [32]) and found that performance improved with some, but not all non-linear methods, with the best performance increase achieved by Support Vector Regression, Dense Neural Network, and LSTM (supplementary figure 4). However, the improvement is typically minimal, confined mostly to well-decoded DoFs, and may not justify the added computational complexity and potential for overfitting. Note, however, that an algorithm that performs better offline does not necessarily perform better online [54, 55].

### 4.6. Closed-loop robotic limb control

A major application of kinematic decoders is to drive brain machine interfaces aimed at restoring movement in patients with sensorimotor impairments (Bensmaia & Miller 2014). Indeed, intended movements can be inferred from neural signals in sensorimotor cortex and converted into control commands to an external device, such as a robotic limb. While remarkable control has been previously achieved for a robotic arm and hand, with up to 10 degrees of freedom under independent control [8], the control of the hand itself remains relatively primitive, restricted to few of its many potential degrees of freedom. Here, we show that information about up to 30 degrees of freedom can be simultaneously reconstructed using a fast and simple approach with a relatively small number of neurons. Note, however, that the decoding reported here was performed offline and the implications of the present results for online decoding need to be tested [32, 54, 55, 71, 72].

The dexterity of robotic hands is severely limited by the absence of sensory feedback about hand posture. One approach to convey proprioceptive feedback would be to stimulate proprioceptive neurons in SC [35–37]. Our results suggest that SC—particularly area 3 a—carries a faithful representation of hand posture, one that could in principle be exploited to convey intuitive sensory feedback about limb state. However, the success of tactile feedback through intracortical microstimulation (ICMS) has hinged on the somatotopic organization of cutaneous representations in SC. While proprioceptive representations exhibit some somatotopic organization [23, 28, 73, 74], that organization is generally coarser than that seen in tactile representations in cortex and may depend on whether movements are actively generated or imposed on the limb [29, 75]. Thus, whether the proprioceptive representation exhibits a spatial topography that can be exploited to convey artificial proprioceptive feedback remains to be established.
